# Toward Self-Driven Autonomous Material and Device Acceleration Platforms (AMADAP) for Emerging Photovoltaics Technologies

**DOI:** 10.1021/acs.accounts.4c00095

**Published:** 2024-04-23

**Authors:** Jiyun Zhang, Jens A. Hauch, Christoph J. Brabec

**Affiliations:** † Forschungszentrum Juelich GmbH, Helmholtz-Institute Erlangen-Nürnberg (HI ERN), Department of High Throughput Methods in Photovoltaics, Immerwahrstraße 2, 91058 Erlangen, Germany; ‡ Friedrich-Alexander-University Erlangen-Nuremberg, Faculty of Engineering, Department of Material Science, Institute of Materials for Electronics and Energy Technology (i-MEET), Martensstrasse 7, 91058 Erlangen, Germany

## Abstract

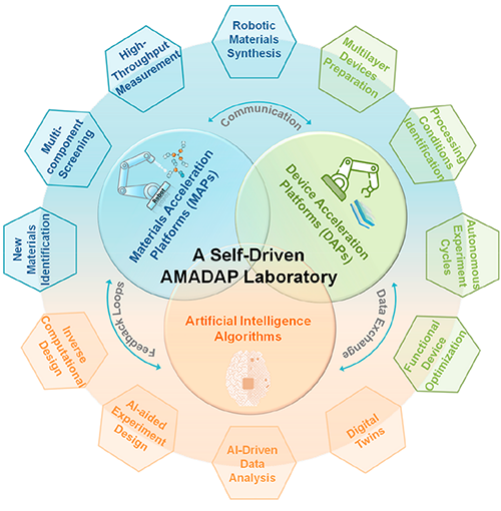

In the ever-increasing renewable-energy demand scenario, developing new photovoltaic technologies is important, even in the presence of established terawatt-scale silicon technology. Emerging photovoltaic technologies play a crucial role in diversifying material flows while expanding the photovoltaic product portfolio, thus enhancing security and competitiveness within the solar industry. They also serve as a valuable backup for silicon photovoltaic, providing resilience to the overall energy infrastructure. However, the development of functional solar materials poses intricate multiobjective optimization challenges in a large multidimensional composition and parameter space, in some cases with millions of potential candidates to be explored. Solving it necessitates reproducible, user-independent laboratory work and intelligent preselection of innovative experimental methods.

Materials acceleration platforms (MAPs) seamlessly integrate robotic materials synthesis and characterization with AI-driven data analysis and experimental design, positioning them as enabling technologies for the discovery and exploration of new materials. They are proposed to revolutionize materials development away from the Edisonian trial-and-error approaches to ultrashort cycles of experiments with exceptional precision, generating a reliable and highly qualitative data situation that allows training machine learning algorithms with predictive power. MAPs are designed to assist the researcher in multidimensional aspects of materials discovery, such as material synthesis, precursor preparation, sample processing and characterization, and data analysis, and are drawing escalating attention in the field of energy materials. Device acceleration platforms (DAPs), however, are designed to optimize functional films and layer stacks. Unlike MAPs, which focus on material discovery, a central aspect of DAPs is the identification and refinement of ideal processing conditions for a predetermined set of materials. Such platforms prove especially invaluable when dealing with “disordered semiconductors,” which depend heavily on the processing parameters that ultimately define the functional properties and functionality of thin film layers. By facilitating the fine-tuning of processing conditions, DAPs contribute significantly to the advancement and optimization of disordered semiconductor devices, such as emerging photovoltaics.

In this Account, we review the recent advancements made by our group in automated and autonomous laboratories for advanced material discovery and device optimization with a strong focus on emerging photovoltaics, such as solution-processing perovskite solar cells and organic photovoltaics. We first introduce two MAPs and two DAPs developed in-house: a microwave-assisted high-throughput synthesis platform for the discovery of organic interface materials, a multipurpose robot-based pipetting platform for the synthesis of new semiconductors and the characterization of thin film semiconductor composites, the SPINBOT system, which is a spin-coating DAP with the potential to optimize complex device architectures, and finally, AMANDA, a fully integrated and autonomously operating DAP. Notably, we underscore the utilization of a robot-based high-throughput experimentation technique to address the common optimization challenges encountered in extensive multidimensional composition and parameter spaces pertaining to organic and perovskite photovoltaics materials. Finally, we briefly propose a holistic concept and technology, a self-driven autonomous material and device acceleration platform (AMADAP) laboratory, for autonomous functional solar materials discovery and development. We hope to discover how AMADAP can be further strengthened and universalized with advancing development of hardware and software infrastructures in the future.

## Key References

- ZhaoY.; HeumuellerT.; ZhangJ.; LuoJ.; KasianO.; LangnerS.; KupferC.; LiuB.; ZhongY.; EliaJ.; OsvetA.; WuJ.; LiuC.; WanZ.; JiaC.; LiN.; HauchJ.; BrabecC. J.A bilayer conducting polymer structure for planar perovskite solar cells with over 1,400 h operational stability at elevated temperatures. Nat. Energy2022, 7, 144–15210.1038/s41560-021-00953-z.^1^*Screening of 160 perovskite compositions for photothermal-stable perovskites using a robotic platform. The unsealed perovskite device based on the optimum composition retains 99% peak efficiency after 1450 h of continuous aging conditions.*
- WuJ.; ZhangJ.; HuM.; ReiserP.; TorresiL.; FriederichP.; LahnL.; KasianO.; GuldiD. M.; Pérez-OjedaM. E.; BarabashA.; Rocha-OrtizJ. S.; ZhaoY.; XieZ.; LuoJ.; WangY.; SeokS. I.; HauchJ. A.; BrabecC. J.Integrated System Built for Small-Molecule Semiconductors via High-Throughput Approaches. J. Am. Chem. Soc.2023, 145, 16517–1652510.1021/jacs.3c0327137467341 PMC10401720.^2^*An integrated automatic system was developed for high-throughput synthesis, purification, and characterization of small conjugated molecules. A material library containing 125 new molecules with their optical-electronic properties was built in weeks.*
- ZhangJ.; LiuB.; LiuZ.; WuJ.; ArnoldS.; ShiH.; OsterriederT.; HauchJ. A.; WuZ.; LuoJ.; WagnerJ.; BergerC. G.; StubhanT.; SchmittF.; ZhangK.; SytnykM.; HeumuellerT.; Sutter-FellaC. M.; PetersI. M.; ZhaoY.; BrabecC. J.Optimizing Perovskite Thin-Film Parameter Spaces with Machine Learning-Guided Robotic Platform for High-Performance Perovskite Solar Cells. Adv. Energy Mater.2023, 13, 230259410.1002/aenm.202302594.^3^*An ML-driven automated platform, SPINBOT, efficiently explored a complex parameter space of perovskite thin films. The solar cells based on the optimized film achieved an impressive 21.6% efficiency under ambient conditions, along with excellent long-term stability.*
- OsterriederT.; SchmittF.; LüerL.; WagnerJ.; HeumüllerT.; HauchJ.; BrabecC. J.Autonomous optimization of an organic solar cell in a 4-dimensional parameter space. Energy Environ. Sci.2023, 16, 3984–399310.1039/D3EE02027D.^4^*By a combination of the AI algorithm and automatic system, we achieved AI-guided closed-loop autonomous optimization for fully organic solar cells in a 4-dimensional parameter space.*

## 1 Introduction

In recent decades, the shift toward a renewable clean energy scenario to further reduce greenhouse gas emissions has driven a rather novel research direction utilizing automated and robot-assisted laboratories.^5^ These laboratories are expected to accelerate new materials discovery, such as functional solar materials, which convert solar energy into electricity.^6,7^ However, in the field of photovoltaics or, more generally, in the field of optoelectronics, the discovery of a new material for a functional optoelectronic device typically takes decades. For example, it took silicon several decades to transition from the discovery of the photovoltaic effect in silicon in 1941 to the demonstration of the first silicon solar cell in 1954 all the way to the late 1970s to establish a production capacity of about 1 MW/year.^8,9^ A similar timeline can be found for electroluminescence in gallium nitride (GaN) and the deployment of light-emitting diodes based on GaN.^10^ For as long as scientific methodologies have been developed, researchers have desired faster, dependable, and more efficient methods of experimentation for new materials discovery. This desire was probably best expressed in the introduction of the “Materials Genome Initiative (MGI)” under the Obama legislation in 2011.^11,12^ The announcement of the MGI led the academic community to expedite the pace of advanced materials discovery, development, manufacture, and deployment in a more high-throughput and economical manner.^13^

Autonomous acceleration research platforms have been introduced to explore the realms of materials science research and development (R&D).^14,15^ While the MGI expressed an acceleration of a factor of 2 and a reduction of costs by a factor of 2, some of the modern MAPs are proposing larger acceleration factors, ideally reducing the entire materials R&D cycle from typically 10 to 20 years to only 1 or 2 years.^5,16^ Such acceleration factors become possible when automated laboratory setups are equipped with artificial intelligence (AI).^17,18^ These setups build on recent scientific breakthroughs and the ability to program machines so that they can make independent and autonomous decisions to design and optimize materials and processes, moving away from the traditional Edisonian method of discovery.^19,20^ Such breakthroughs hold the promise of a true digital twin of a technology, allowing science to predict optimized materials and processes according to the requirements of a target application.^21,22^ The methodology of integrating automation and autonomous operation into emerging photovoltaics research, such as perovskite solar cell (PSC) and organic photovoltaic (OPV) materials, is drawing broad attention from the community.^3,4,23−27^ MAPs and DAPs offer tremendous opportunities for accelerating functional materials discovery and device optimization with respect to performance, environmental stability, and cost.

Herein, we report the recent progress of our group in exploring MAPs and DAPs with a primary emphasis on emerging photovoltaic materials and devices, for example, perovskite and organic photovoltaics. We present the design of various self-driven laboratories and report acceleration factors for specific research quests obtained along these lines. We rationalize the strategy behind developing four separated automated lines, followed by stepwise introduction into the current concepts and technologies to accelerate emerging photovoltaics development. The outlook provides the concept and vision to fuse MAPs with DAPs and generate an AMADAP that holds the potential to develop a digital twin with inverse predictive power for emerging photovoltaics.

## 2 Autonomous Materials and Device Acceleration Platforms (AMADAP) for Emerging Photovoltaics

The original design for the various platforms followed the idea to design an automated workflow covering (1) the synthesis of organic as well as inorganic semiconductors, (2) ink design, composite engineering, and film formation, (3) device processing of organic and perovskite semiconductors under environmental conditions, and (4) device processing and characterization of organic and perovskite semiconductors in a nitrogen environment. The historic development of automated platforms did not follow this sequence but started in 2014 with the first installation of a robot-based pipetting platform based on a Tecan Freedom EVO 100 within the project *MatSol* (*SOLARFABRIK DER ZUKUNFT*). One year later, the installation of AMANDA Line One began, supported by a Deutsche Forschungsgemeinschaft (DFG: German Research Foundation)-funded large-scale facility proposal (*DFG 90/917-1 FUGG*). The SPINBOT platform was developed afterward as a versatile multipurpose tool for synthesis, film formation, and device processing and was designed to fit into standard size hoods. After licensing the SPINBOT concept to the company SCIPRIOS, it was continuously expanded with multiple coating techniques and more advanced characterization routines. The semiautomated platform for organic synthesis is the most recent development (from 2020) and is currently operated in a workflow where samples are moved by hand between the single stages. A decision for transforming this platform into a fully automated laboratory has not yet been made.

### 2.1 Semi-Automated Organic Synthesis Platform

**Figure 1:**
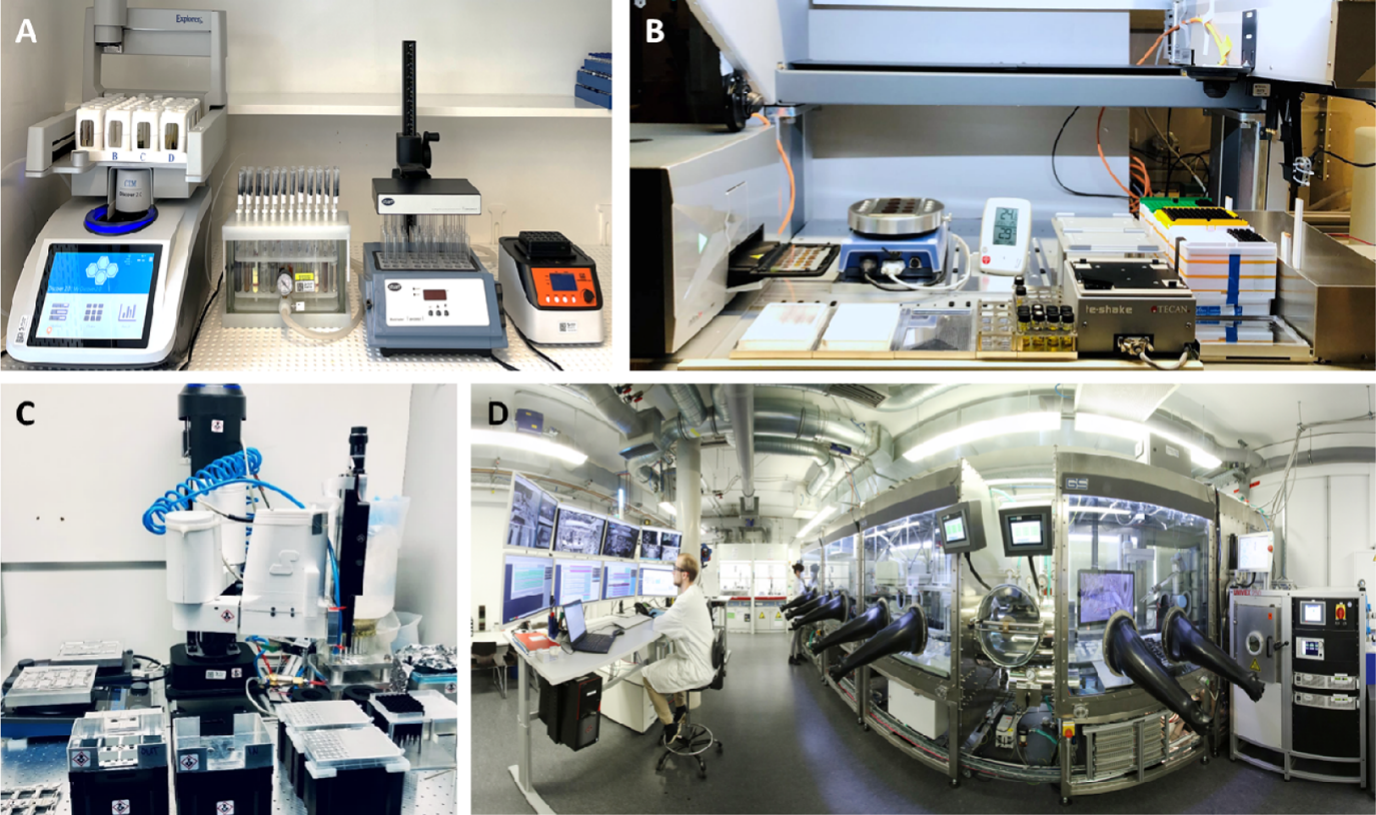
Autonomous materials and device acceleration platforms (AMADAP) within our laboratory. **A.** Semiautomated organic synthesis platform. **B.** Robot-based pipetting platform. **C.** SPINBOT system. **D.** AMANDA platform.

The synthesis system (Figure 1A) is capable of executing three high-throughput (HT) processes: HT syntheses, purification, and characterization. Compared with conventional reflux heating techniques, this modern microwave reactor-based system enhances product yields while dramatically shortening reaction times from days to mere hours or minutes. The reactor operates automatically and can sequentially process up to 48 individual reactions.^28^ This organic semiconductor synthesis platform and the corresponding workflows were developed with the aim of gaining an understanding of the purity of organic semiconductors synthesized with MIDA-type Suzuki coupling click reactions.^2^ Efficiently and securely handling the purification workflow, including solution distribution, heating, stirring, and cooling for screening the optimal solvent composition for product recrystallization, was a major focus.

### 2.2 Robot-Based Pipetting Platform

The robot-based pipetting platform is based on a Tecan Freedom EVO 100 setup and is used in our laboratories for multicomponent precursor preparation, film coating, and characterization as shown in Figure 1B. This platform enables the preparation and screening of an extensive array of candidates, encompassing hundreds of possible components. Different coating processes for organic or perovskite semiconductors, such as drop-casting or spin-coating, were implemented to handle a scalable number of mixable components and a number of films. The largest campaign on this platform required processing over 6000 organic semiconductor films from various recipes and hundreds of inks per day.^29^ To match the unique production capacity, an in-house HT characterization system is coupled to carry out optical measurements, including recording the UV–vis absorption, steady-state photoluminescence (PL), and time-resolved photoluminescence (TRPL) spectra.

### 2.3 SPINBOT Platform

Figure 1C presents the SPINBOT platform. The robot arm used for movement along four different axes (*X*, *Y*, *Z*, and *R* axes) is a selective compliance assembly robot arm (SCARA) with high speed, flexibility, and rigidity. The robot can perform multiple selective tasks in a broad research field repetitively with high accuracy, efficiency, and precision required for automatic solution-processed thin films and device processing. Over 140 thin films per hour with variable processing parameters can be obtained with a high level of processing diversity and reproducibility in air. This platform supports various film and device production processes, in particular, perovskites, including antisolvent deposition, two-step sequential deposition, gas-quenching-assisted, and hot-casting methods.

### 2.4 AMANDA: An Autonomous Device Acceleration Platform

AMANDA is (Figure 1D) controlled by a self-developed software backbone specifically designed to fabricate and characterize solution-processed thin film devices, for example, OPVs. It can perform accurate closed-loop screenings of up to 272 sample variations (∼1632 solar cells) per day.^30^ Each solar cell is fully characterized by photography, UV–vis absorption, electrode evaporation, and current-density versus voltage (*J*–*V*) measurements, and all process steps are comprehensively documented. The whole process can be fully automated with multiple tasks being performed in parallel and has been recently set to autonomous operation, requiring no further human intervention to find optimized device processing conditions.

## 3 Autonomous MAPs for Emerging Photovoltaics Technologies

In recent years, progressive advances to improve both the efficiency and stability of solution-processed OPVs and PSCs have been achieved by compositional and processing engineering, which points out a clear trend toward multicomponent active-layer blends.^31−34^ On the pathway to gain predictive power for the discovery of new materials or optimized composites for performance enhancement, the need for innovative experimental methods becomes increasingly urgent. Robot-based high-throughput experimentation (HTE) effectively addresses various challenges and is already providing powerful tools to the scientific community to effectively screen libraries of several thousands to tens of thousands of candidates.^35^

### 3.1 Discovery of New Hole-Transporting Materials

**Figure 2:**
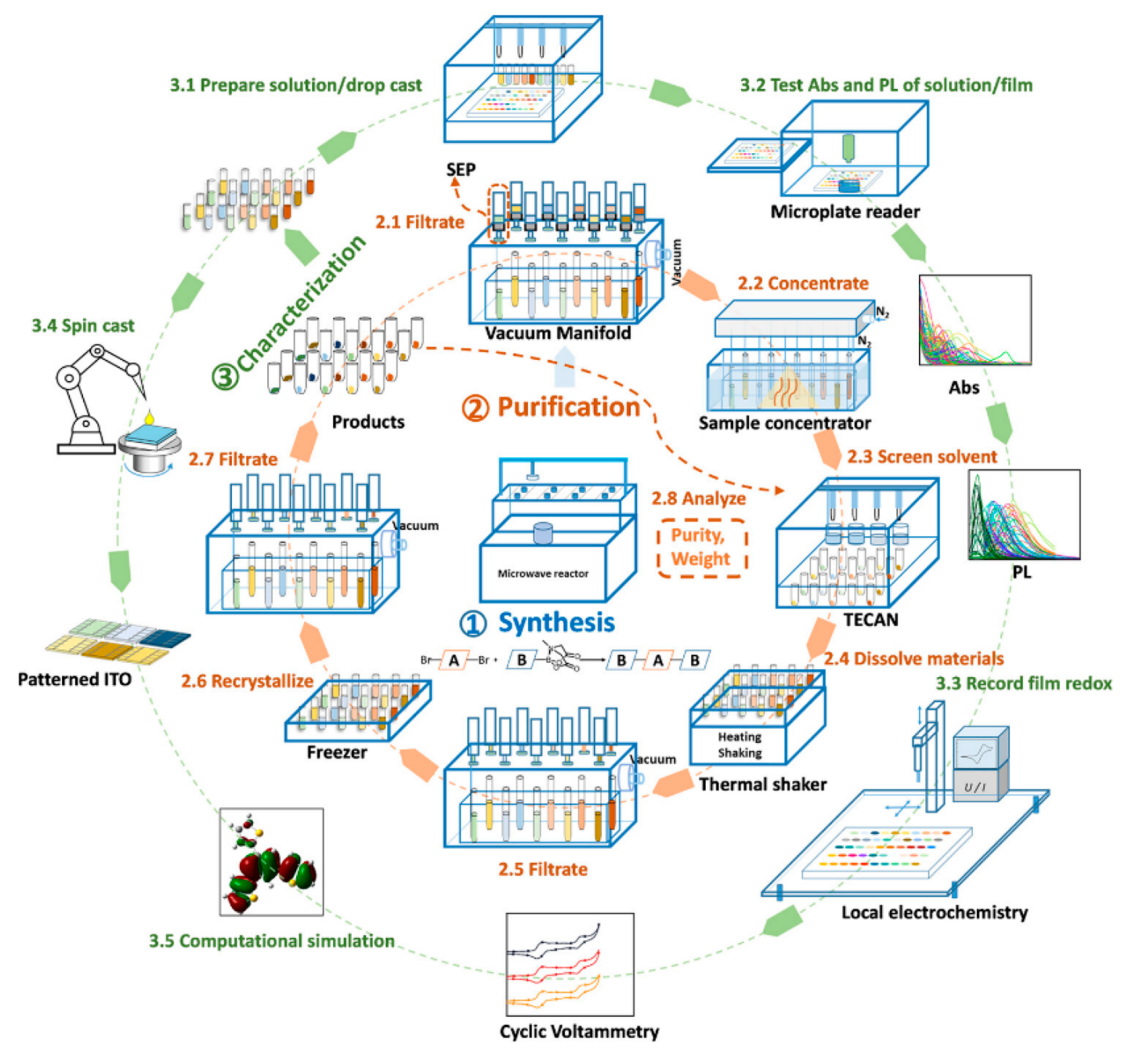
Workflow of the robot-based high-throughput synthesis, purification, and characterization of conjugated small molecules. Reproduced with permission from ref (2). Copyright 2023, American Chemical Society.

Conjugated small molecules have been widely used in many functional semiconductor applications such as PSCs,^36^ OPVs,^37^ and organic light-emitting diodes.^38^ Building a comprehensive material library and property database for conjugated molecules has the potential to expedite the exploration of broadly applicable structure–property relationships and accelerate the discovery of new molecules beyond current rules. Probably the biggest challenge is the purity and electronic quality of molecules synthesized by click reactions such as the MIDA library. To clarify this question, we developed an integrated workflow, the “organic semiconductor synthesis platform”, for the synthesis of organic small molecules comprising three automatic HT processes: synthesis, purification, and characterization.^2^ As shown in Figure 2, the workflow was heavily focused on automated purification and characterization. The first step involves microwave-assisted Suzuki–Miyaura coupling reactions with monomers from the MIDA library. The MIDA protected monomers were primarily chosen due to their binary elution properties and affinity for silica gel, easing the development of an automated purification process. Optimized processing conditions provided yields of up to 95% at reaction times of less than an hour. For purification, a two-step process using filtration and recrystallization was employed and repeated until the materials achieved sufficient purity of over 70%. The purified materials were then characterized through various techniques, including absorption, PL, cyclic voltammetry (CV), and conductivity, and were completed by computational simulations. As a result, the semiautomatic organic synthesis platform allowed us to compile a fully characterized material library containing 125 conjugated small molecules together with their optoelectronic properties within a time frame of weeks. The availability of such material libraries is essential to scientists across various fields in order to train predictive models such as Gaussian process regression (GPR) that assists the establishment of structure–property–performance relationships but also accelerates the discovery of novel molecules. The first candidates from this library are currently screened as hole transport materials (HTM) in perovskite cells and already show better performance than the commonly used poly[bis(4-phenyl)(2,4,6-trimethylphenyl) amine (PTAA) material, the gold standard for HTM screening for p-i-n perovskite architectures. The demonstration of highly performing perovskite cells with HTMs synthesized with Suzuki–Miyaura coupling of MIDA protected boronates is an impressive demonstration that this route can yield electronic-grade materials without high-performance liquid chromatography (HPLC) purification. In the next step, we plan to use the HTM library to train a Gauss process capable of predicting the power conversion efficiency (PCE) of PSCs at the hand of the HTM molecular descriptors.

### 3.2 High-Throughput-Based Antisolvent Crystallization of Perovskites

Antisolvent crystallization strategies are commonly used to fabricate high-quality perovskite materials.^39^ Although crucial, there is currently a limited exploration of the fundamental chemistry involved in solvent and antisolvent crystallization, and a comprehensive understanding is lacking in this field. Our group, for the first time, utilized a robot-based pipetting platform to rapidly screen the potential antisolvents for various solvent–perovskite systems.^40^ 48 organic liquids were chosen to serve as antisolvents and categorized based on their dielectric constant (ε). In total, the integrated platform synthesized and characterized 336 different combinations of perovskite/solvent/antisolvent within just 2 days. The results indicate that the nature of halogen atoms significantly influences the formation of intermediate complexes, in addition to the well-established impact of solvents. The effectiveness of a solvent in serving as an antisolvent hinged on its ability to disrupt the coordinative bonds between solvent molecules and the central Pb^2+^. That allowed us to successfully elucidate the complex mechanisms underlying solvent/antisolvent crystallization and compile an extensive list of potential antisolvents, thus obviating the necessity for laborious trial-and-error testing. This significantly enhances the production of high-performance perovskite-based materials. As an example, to develop stable wide-band-gap perovskites, 95 different metal halide perovskite samples were efficiently synthesized based on antisolvent crystallization and characterized through the robotic pipetting platform.^41^ This effort yielded six promising compositions with an optical band gap of approximately 1.75 eV. These compositions, including Cs–MA (Cs, cesium; MA, methylammonium), MA–FA (FA, formamidinium), and Cs–FA systems, exhibit excellent photostability, with maximum open-circuit voltage values reaching 1.18, 1.19, and 1.14 V, respectively. This demonstrates the effectiveness of robot-based HTE in expediting the development of novel and efficient perovskites for advanced optoelectronic applications.

### 3.3 Intercalating-Cation Engineering for Stable Quasi-2D Perovskites

Perovskite materials have been widely reported to suffer from intrinsic instability and degradation that hinder their practical applications. The introduction of large organic cations to form quasi-2D perovskites has shown intriguing promise in stabilizing 3D-networking perovskites. The specific type and doping concentration of these cations remain areas that require systematic study to understand the stability of reduced-dimensional perovskite films.^42,43^ Recently, we investigated the long-term thermal stability of a series of Ruddlesden–Popper-type perovskites with different organic cations by preparing and characterizing hundreds of multicomponent films using the robotic pipetting platform. The results show that longer-chain linear-alkyl-ammonium cations in films hindered Ostwald ripening during thermal aging due to a stronger steric-hindrance effect between adjacent perovskite domains. Shorter-chain cations promoted increased-dimensional phase redistribution, aiding the regeneration of 3D/3D-like perovskite phases and sustaining superior stability.^44^ Surprisingly, within the aromatic-based cation system, films with longer-chain phenylpropylammonium cations exhibited reduced stability even compared to shorter-chain ones, primarily due to lattice distortion and phase mismatch. On the other hand, films containing phenylbutanammonium cations displayed robust stability, attributed to distinctive crystallization kinetics and a gradual phase transformation (from larger to smaller phases) driven by steric hindrance.^45^ We found that approximately 20–25 mol % (nominal *n* = 4–5) cation intercalation into MAPbI_3_ can maximize the film stability, while higher or lower concentrations lead to inferior stability, which is termed stability bowing by analogy to band gap bowing.^46^ These works highlighted the complex effect of the type, concentration level, and steric hindrance of intercalating cations on the perovskite stability performance.

### 3.4 High-Throughput-Based Composition Screening of Stable Perovskite Materials

**Figure 3:**
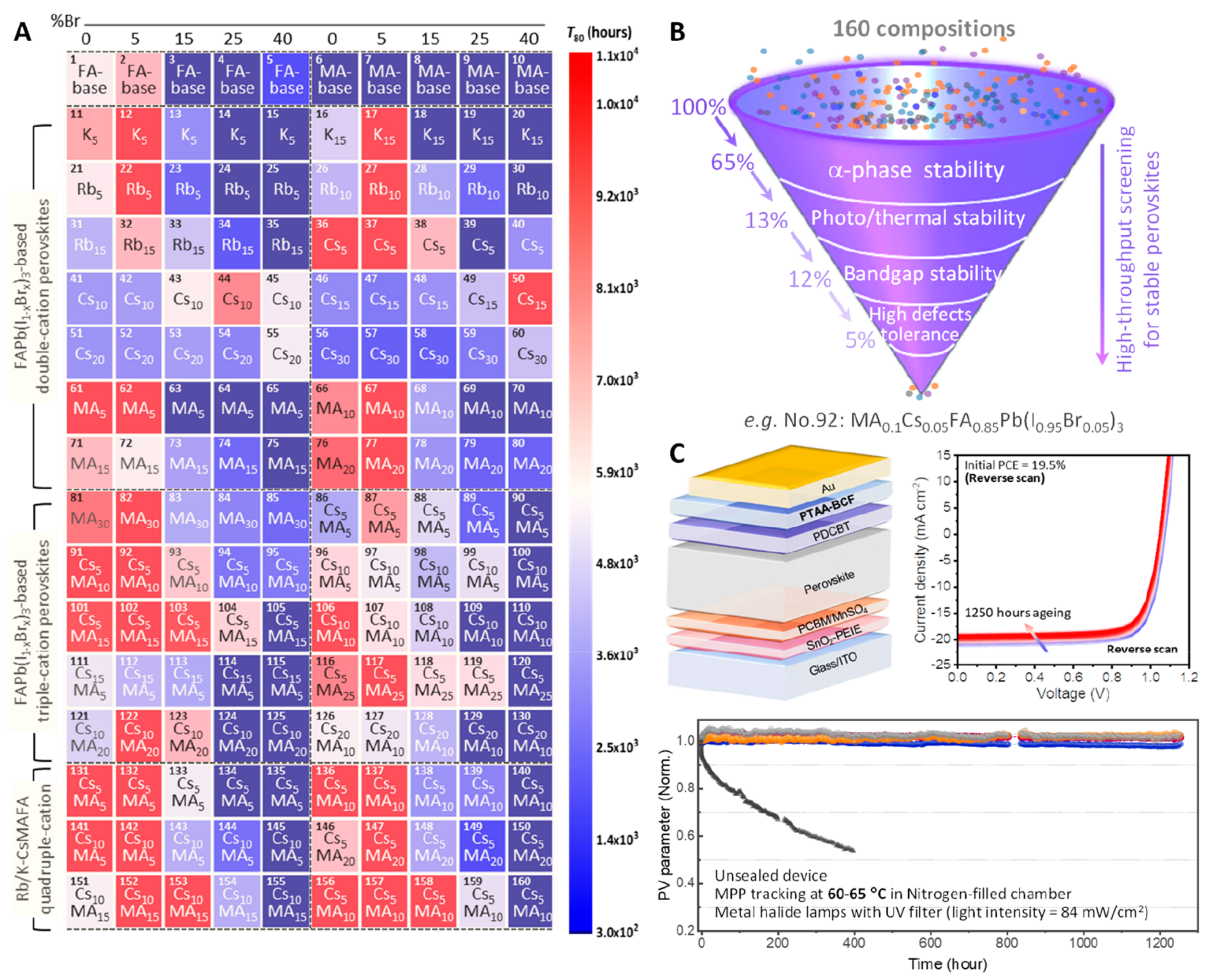
**A.** Color map of *T*_*80*_ lifetimes for the 160 perovskites aged at 65 °C under 100 mW/cm^2^ metal halide light illumination in N_2_-filled chambers. **B.** The robot-based screening process for photothermal-stable perovskite compositions. **C.** Schematic of the solar cell structure, *J*–*V*, and photothermal stability performance for the device with optimum perovskite composition. Adapted with permission from ref (1). Copyright Nature Springer 2021.

Compositional engineering is crucial for the achievement of highly stable perovskites, but traditional experimental methods involving manual tasks can be time-consuming and prone to data inaccuracies. The robot-based pipetting platform allows us to reveal the stability mechanisms of metal halide perovskites by accurately preparing and collecting data of numerous samples with diverse components.^47^ Through the integration of automated HTE with a machine-learning (ML) algorithm, we discovered a correlation between high- and low-temperature stability and identified a stability-reversal behavior.^25^ The influence of a specific cation on stability can jump between detrimental and beneficial when the aging condition alternates between low and high temperatures. For instance, doping at least 10 mol % organic MA and up to 5 mol % inorganic Cs/Rb (Rb, rubidium) cations into the perovskite lattice is recommended to enhance the stability of perovskites at temperatures below 100 °C. The mechanism underlying the stability reversal is the change in both activation energy and rate constant in the decomposition by doping multiple cations into the perovskite lattice. An in-depth HT investigation into compositional engineering in I/Br mixed perovskites is expected to unveil more effective strategies to improve the perovskite stability. To this end, we further utilized the robotic-based pipetting platform to screen an additional 160 perovskite compositions for photothermal-stable perovskites (Figure 3A).^1^ As shown in Figure 3B, the screening process serves as a funnel for the 160 perovskites, allowing only exceptional perovskites to pass through the screening process under stringent selection criteria.

From the statistical analysis, we found that the optimal concentration for Br/K/Rb/Cs (K, potassium) was around 5 mol %, and the doping of MA did not show detrimental effects on the stability. Most photothermal-stable perovskites contain 5 mol % Br and approximately 10 mol % MA, indicating a generally positive influence on the photothermal stability of multicationic, mixed-halide perovskites at temperatures below 100 °C. When the learned knowledge was translated to PSC application, the unsealed bilayer contact device based on the optimum cation composition retained 99% of its peak efficiency after 1,450 h of continuous operation at 65 °C in an N_2_ atmosphere under metal halide lamps (Figure 3C).

### 3.5 Autonomous Multicomponent Screening of the Photostable Organic Active Layer

**Figure 4:**
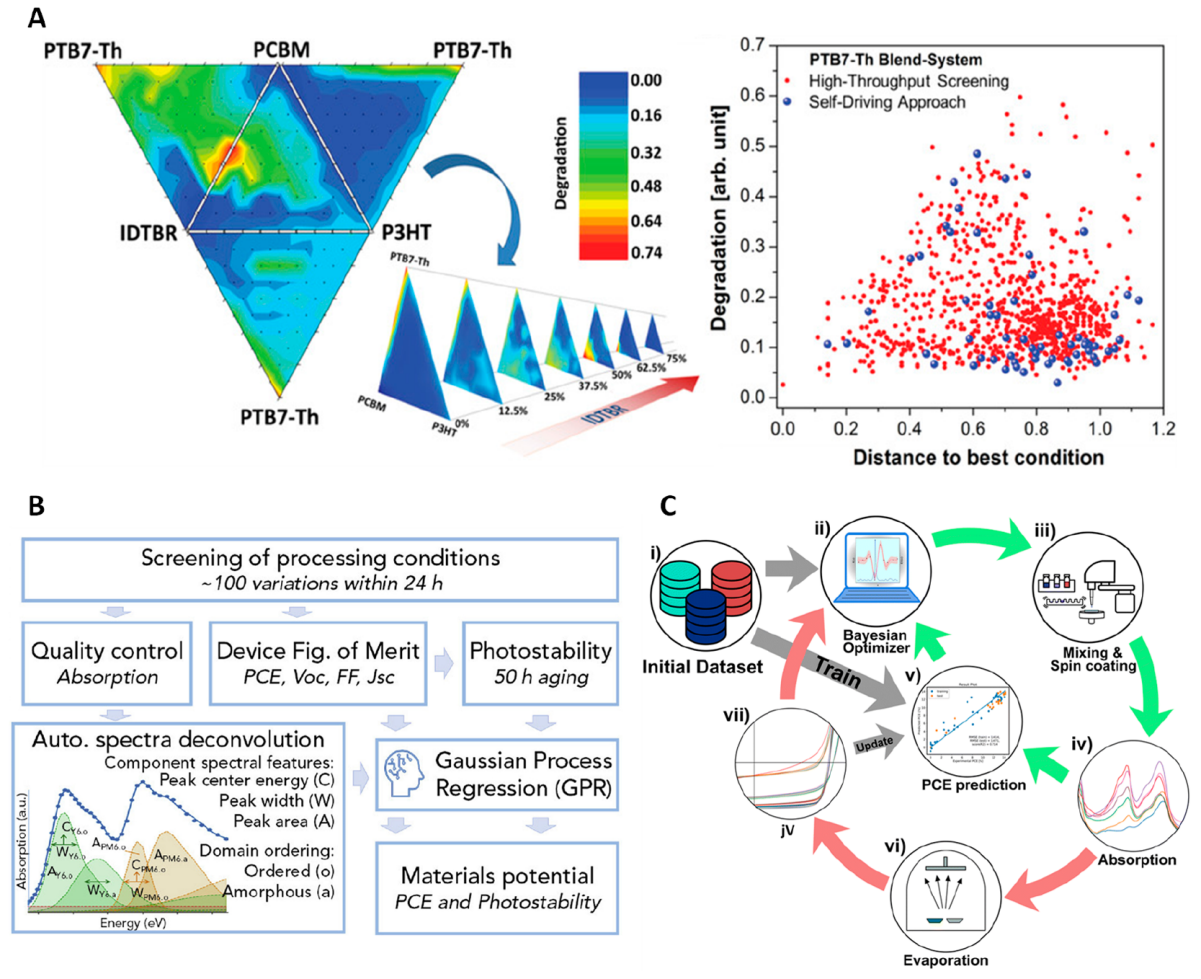
**A.** Photostability of the quaternary system. Degradation is defined as the relative spectral loss in absorbance. Comparison of the covered experimental space between grid-based HTE and self-driving optimization. Reproduced with permission from ref (29). Copyright 2020, Wiley. **B.** Workflow for evaluating OPV materials in terms of efficiency and photostability with GPR-based data analysis. Reproduced with permission from ref (26). Copyright 2021, Elsevier. **C.** Schematic of ML-guided closed-loop optimization using UV–vis data to predict the PCE of organic solar cells. Reproduced with permission from ref (4). Copyright 2023, Royal Society of Chemistry.

Designing reliable multicomponent active-layer blends is a key step in achieving high efficiency and stable OPVs. However, effectively exploring a multidimensional space of thousands of possible composites and additive candidates comes with many challenges. The first is to enable the rapid and scalable manufacturing of high-quality individual layers. The automatic pipetting platform allowed us to produce up to 6048 individual active layers per day and characterize up to 2000 films within just 1 week, requiring less than 15 mg of material per composition, which far exceeds traditional experimental planning and evaluation capabilities. As an experimental proof of concept, a 4D parameter space of quaternary organic solar cell (OSC) blends was created and optimized (Figure 4A).^29^ The high-throughput-based experimentation smoothly completed the preparation and stability evaluation of thousands of multicomponent active layers and revealed their interactions with other components. To accelerate the multicomponent screening of active layers for photostable OPVs, the automated MAP was integrated with an ML tool to form a self-driving laboratory. Through this ML-driven combinatorial approach, stability data of thousands of multicomponent active layers were analyzed, making it possible to extrapolate the results of all possible experiments based on the resulting statistical correlation information. The findings illustrated the potential to enhance the photostability of OPVs through screening a space of thousands of potential composites enabled by autonomous experimentation, which paved the way for achieving more stable and efficient solar energy technologies.

## 4 Multidimensional Parameter Space Optimization of Perovskite and Organic Solar Cells with DAPs

Similar to the critical role played by the optimal selection of active layer and interface layer materials, device optimization itself is equally decisive in influencing the performance of photovoltaics. However, the development of complex functional devices poses a multiobjective optimization problem in a large high-dimensional parameter space. Solving it requires reproducible, user-independent laboratory work and an intelligent preselection of experiments. Moreover, one realizes that accelerating a whole technology requires more than accelerated materials research; that is, it also takes devices and process development to truly accelerate the emerging photovoltaic technologies. Device acceleration platforms offer a promising solution for device and processing development.

### 4.1 Organic Photovoltaics Materials

Optimizing the high-dimensional processing space for bulk-heterojunction OPVs presents significant importance for enhancing both PCE and stability performance. The AMANDA system, a self-developed DAP in our laboratory, has achieved a high level of maturity and enables fully automated processing of hundreds of OSCs with great speed, accuracy, and efficiency.^30^ By following an experiment-as-a-service model, this platform streamlines experiment design from execution and allows researchers to mainly focus on data evaluation and mental efforts, such as conception design. To achieve a multitarget evaluation, this DAP enabled exploring over 100 process conditions of OPV materials at the full-device level, which contributed to forming a high-quality data set.

By coupling the GPR prediction based on cost-effective proxy measurements, such as optical absorption features, the data set enabled promising predictions for both efficient and photostable OPV devices (Figure 4B).^26^ The combination approach demonstrated the huge potential of accelerated development of high-performance solar materials, optimal process parameters, and device structures with a lower input cost.

Building on this foundation, a pioneering concept of AI-guided closed-loop autonomous optimization for fully functional organic devices was then introduced, as shown in Figure 4C. The power of the autonomous approach was exemplified through the optimization of a 4-dimensional parameter space defined by multicomponent and processing parameters for a PM6:Y12:PC70BM-based ternary OPV system.^4^ As a result, the optimal parameter sets for the ternary system and precise objective function across the intricate parameter space were identified using only 40 samples, a significant reduction compared to the approximately 1000 samples required by a traditional Edisonian approach. These studies highlight the significance of integrating robot-based DAP with fitting ML algorithms, leading to the accelerated identification of optimum conditions for high-performance solution-processed devices and ultimately expediting progress in emerging photovoltaics technologies.

### 4.2 Perovskite Photovoltaics Materials

**Figure 5:**
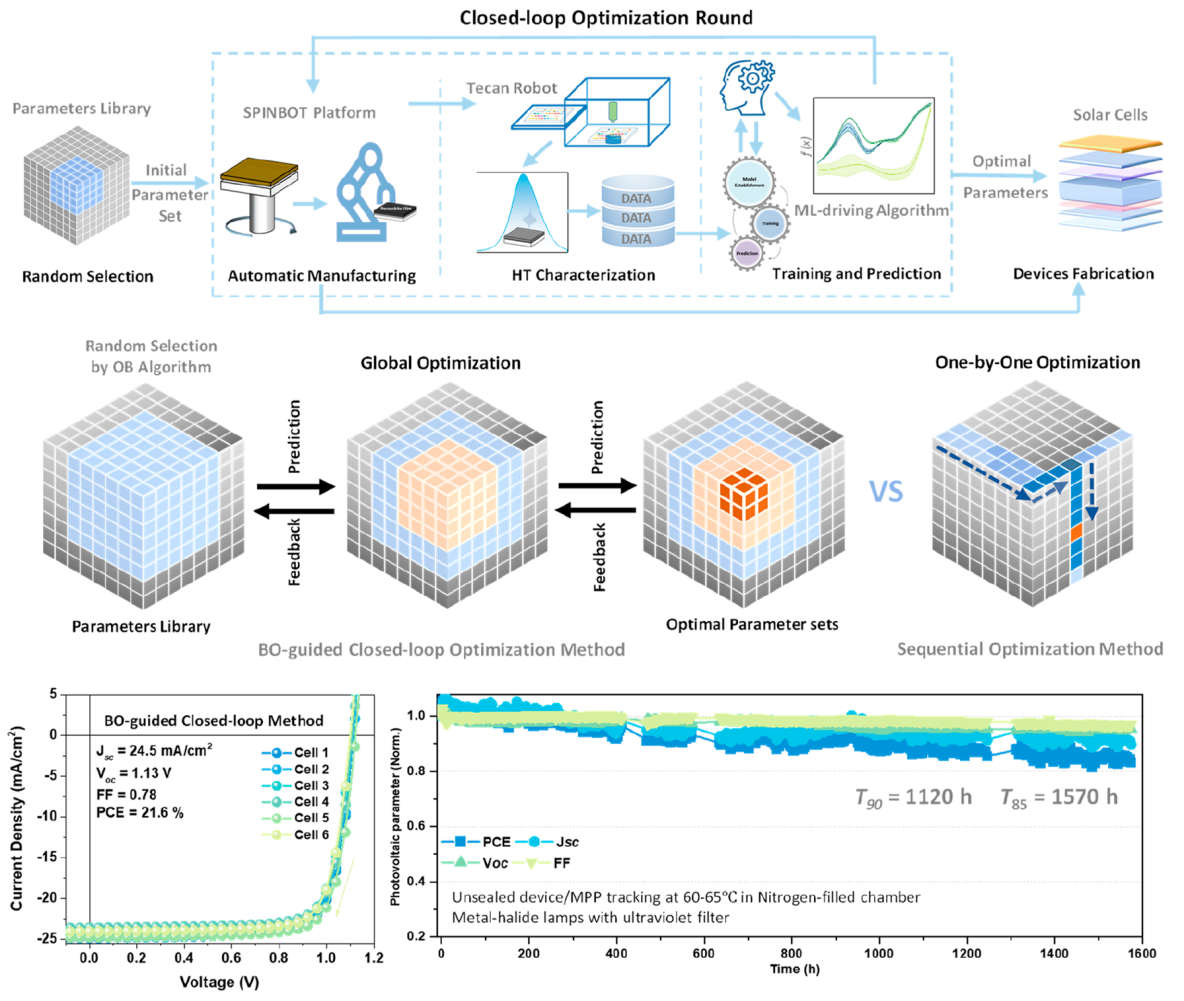
The device acceleration platform, SPINBOT, integrates the ML algorithm to optimize perovskite thin films for high-performance PSCs. It efficiently explores an intricate multidimensional parameter space through the BO-guided closed-loop optimization method. The global optimization method identified the optimal conditions, which led to high-performance PSCs with a champion efficiency of 21.6% in ambient air. Adapted with permission from ref (3). Copyright 2023, Wiley.

In addressing the intricate challenge of simultaneously optimizing manufacturing parameters for perovskite films and devices in air, a fully automated DAP named SPINBOT was developed. To demonstrate the exceptional experimental capabilities, this DAP executed 5 optimization steps including a total of 61 variables for perovskite thin film fabrication. The step-by-step optimization process identified an optimal condition, which resulted in a champion perovskite device with a PCE of 21%.^3^ The exceptional manufacturing process control and data reproducibility offered by this automated DAP were exemplified through repeating device fabrication under optimal conditions. Recognizing the limitations of this approach, such as limited parameter ranges and possible local optimizations, a Bayesian optimization algorithm was then introduced to direct the DAP for global optimization. As a result, the ML-guided DAP effectively explored the complex parameter space containing more than millions of combinatorial parameter sets and enabled continuous improvements in the quality and reproducibility of perovskite thin films. The intelligent closed-loop optimization approach achieved a series of processing parameters that promptly led to perovskite devices with an impressive champion efficiency of 21.6% and satisfactory photothermal stability (Figure 5). The SPINBOT platform facilitates the achievement of high-performance full PSCs under ambient conditions by enabling precise control and optimization of various process parameters, moving beyond traditional approaches. Through DAP, eight process parameters have been systematically optimized. Among the optimized parameters, the dispensing speed of organic ammonium halide stands out due to its complexity and difficulty in manual control. This optimization has led to the establishment of a standard operation procedure (SOP) for additive-free processing of perovskite devices with efficiencies exceeding 23% in ambient air, along with satisfactory performance reproducibility and photothermal stability.^48^ These outcomes underscore the crucial importance of precise process parameter optimization in enhancing the perovskite photovoltaics performance, and they attest to the critical role of automated platforms in streamlining experimental workflows and accelerating high-performance perovskite photovoltaics development.

## 5 Combining MAPs and DAPs into a Self-Driven AMADAP Laboratory

**Figure 6:**
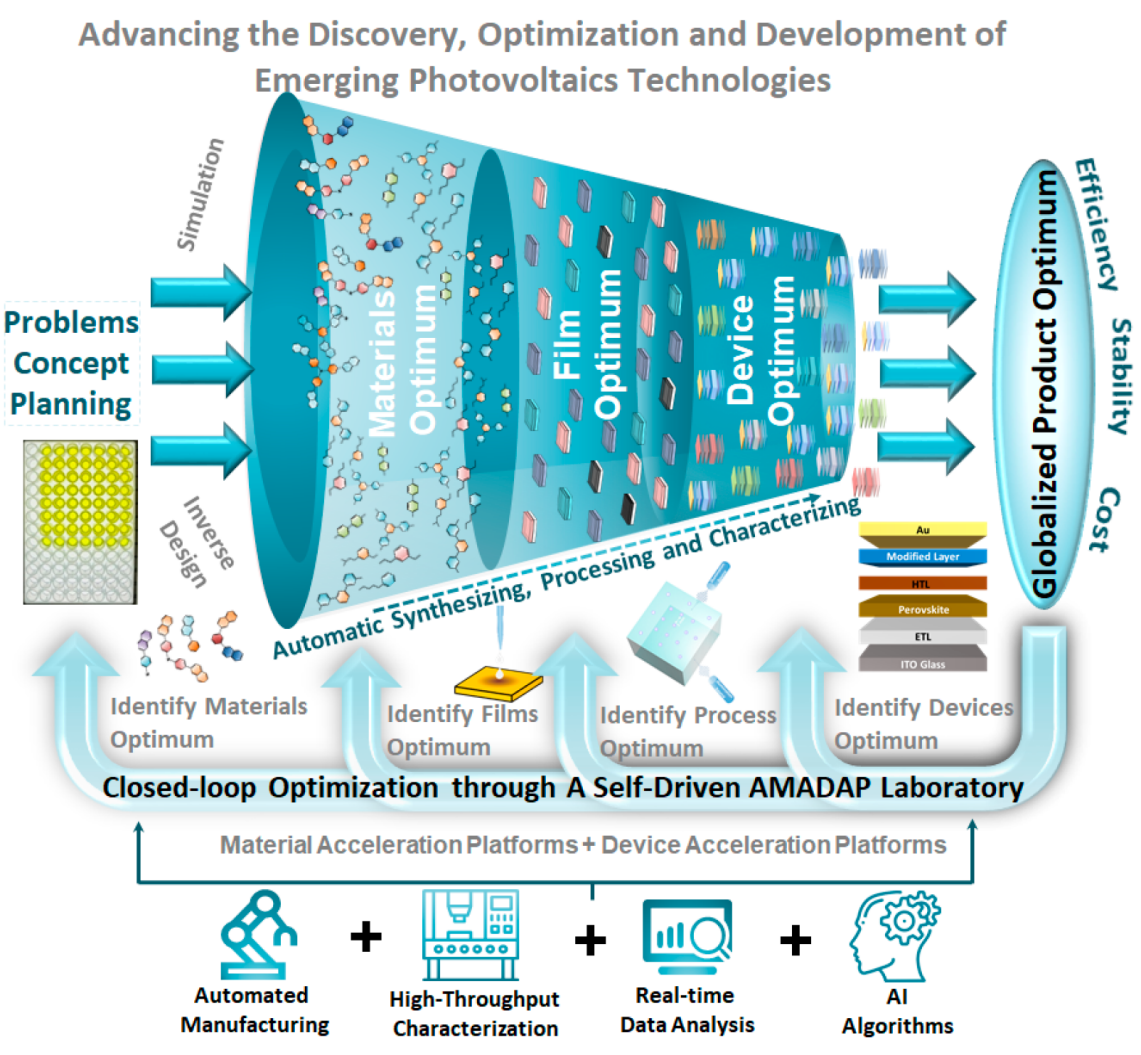
Schematic of a holistic technology: a self-driven autonomous material and device acceleration platform (AMADAP) laboratory. The collaborative synergy between MAPs and DAPs, facilitated by streamlined communication, data exchange, and feedback loops, presents an innovative model for efficiently transitioning from promising materials identification to processing condition refining and multilayer device optimization in the realm of advanced emerging photovoltaics technologies.

The introduction of automation in materials science marks a significant shift in the research paradigm, which lays the foundation for the realization of the “self-driving laboratory” concept.^49−51^ While independent MAPs excel in rapidly screening materials, they often lack the capability to directly translate these findings into optimized devices. Similarly, DAPs are adept at refining device performance but may be isolated from the innovative materials developed in MAPs. In this context, the collaborative combination of MAPs with DAPs offers a pioneering strategy to advance new material discovery and device optimization in the realm of emerging photovoltaics. This innovative approach to streamline communication, data exchange, and feedback loops between MAPs and DAPs can ensure an efficient transition from identifying promising materials to refining processing conditions and optimizing functional devices. The synergy enhances the overall research workflow, which allows for a more systematic and accelerated approach to developing tailor-made functional materials for advanced emerging photovoltaic technologies. The introduction of the AI algorithm and digital twins is essential for optimizing the link between MAPs and DAPs.^22,52^ The digital twins can provide a virtual representation of both materials and devices, which not only enables real-time monitoring, analysis, and simulation of experiments but also enhances the efficiency and reliability of autonomous operation. On the whole, this integrated approach goes beyond autonomous optimization and lead to a paradigm shift toward a self-driven laboratory environment, as exemplified by the proposed concept of autonomous material and device acceleration platforms (AMADAP), as shown in Figure 6. The proposed AMADAP envisions a holistic solution for advancing emerging photovoltaics through the continuous improvement and efficient utilization of hardware and software infrastructures. In the future, the integration of emerging technologies such as blockchain for enhanced data security and integrity and the Internet of Things (IoT) for improved connectivity will play a crucial role in our strategy. These technologies will further fortify the AMADAP framework and make the transition from material discovery to device optimization not just seamless but also more secure and interconnected, thereby reinforcing the foundation for a truly self-driven laboratory environment.

## 6 Challenges and Strategies

The multifaceted expertise required for the self-design and construction of these automated platforms highlights the current barriers due to a lack of integrated automated production streams and especially the necessity for proficiency across multiple disciplines. As automation technology evolves, collaborations and resource sharing within the research community are pivotal for overcoming these hurdles. Additionally, ensuring adaptability and customization of automated platforms to accommodate diverse materials and research goals remains a challenge, which can be addressed through modular designs and adaptable workflows. While significant progress has been made in incorporating various characterization techniques into AMADAP, expanding the range and accessibility of automated high-throughput testing capabilities remains a priority. Ensuring data reliability and achieving platform standardization are also crucial for guaranteeing the comparability and dependability of research outcomes across platforms. Finally, transforming vast amounts of data from automatic platforms to actionable insights requires a robust FAIR data infrastructure, sophisticated data analysis methods, and integrated theoretical and experimental approaches to drive material innovation. Together, these strategies and considerations underscore the ongoing efforts and future directions needed to maximize the potential of AMADAP for accelerating materials discovery and development.

## 7 Conclusions

In addressing the pressing need for advancing photovoltaic technologies, the significance of emerging solar materials and their optimization cannot be overstated. The complex multiobjective optimization challenges of vast composition and parameter spaces demand innovative solutions. This Account emphasizes the role of MAPs and DAPs in tackling these challenges. These platforms represent a paradigm shift from traditional trial-and-error methods to autonomous, ultrashort experimentation cycles with exceptional precision. In particular, this Account highlights in-house-developed MAPs and DAPs and showcases their utility in addressing optimization challenges related to organic and perovskite photovoltaics materials. The introduction of a self-driven autonomous material and device acceleration platform (AMADAP) laboratory concept signals a forward-looking approach to further strengthen and universalize autonomous functional solar materials discovery and development through taking advantage of evolving hardware and software infrastructures. Overall, this Account underscores the pivotal role of advanced automation and autonomy in shaping the future of emerging photovoltaic research and development.
